# Radiomics‐based classification models for HBV‐related cirrhotic patients with covert hepatic encephalopathy

**DOI:** 10.1002/brb3.1970

**Published:** 2020-11-24

**Authors:** Sha Luo, Zhi‐Ming Zhou, Da‐Jing Guo, Chuan‐Ming Li, Huan Liu, Xiao‐Jia Wu, Shuang Liang, Xiao‐yan Zhao, Ting Chen, Dong Sun, Xin‐Lin Shi, Wei‐Jia Zhong, Wei Zhang

**Affiliations:** ^1^ Department of Radiology The Second Affiliated Hospital of Chongqing Medical University Chongqing China; ^2^ GE Healthcare Life Sciences Shanghai China

**Keywords:** cognitive impairment, covert hepatic encephalopathy, neuroimaging, precuneus, radiomics

## Abstract

**Introduction:**

The significant abnormalities of precuneus (PC), which are associated with brain dysfunction, have been identified in cirrhotic patients with covert hepatic encephalopathy (CHE). The present study aimed to apply radiomics analysis to identify the significant radiomic features in PC and their subregions, combine with clinical risk factors, then build and evaluate the classification models for CHE diagnosis.

**Methods:**

106 HBV‐related cirrhotic patients (54 had current CHE and 52 had non‐CHE) underwent the three‐dimensional T1‐weighted imaging. For each participant, PC and their subregions were segmented and extracted a large number of radiomic features and then identified the features with significant discriminative power as the radiomics signature. The logistic regression analysis was employed to develop and evaluate the classification models, which are constructed using the radiomics signature and clinical risk factors.

**Results:**

The classification model (R‐C model) achieved best diagnostic performance, which incorporated radiomics signature (4 radiomic features from right PC), venous blood ammonia, and the Child‐Pugh stage. And the area under the receiver operating characteristic curve values (AUC), sensitivity, specificity, and accuracy values were 0.926, 1.000, 0.765, and 0.848, in the testing set. Application of the radiomics nomogram in the testing set still showed a good predictive accuracy.

**Conclusions:**

This study presented the radiomic features of the right PC, as a potential image marker of CHE. The radiomics nomogram that incorporates the radiomics signature and clinical risk factors may facilitate the individualized prediction of CHE.

## Graphical abstract

Large amount radiomic features of PC and its subregions was significantly different among CHE and nCHE patients. Radiomic features from right PC were effective in distinguishing CHE from nCHE patients. Integrating the radiomics data and clinical risk factors has the ability to improve diagnostic performance of the classifier.

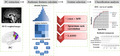

## 1 INTRODUCTION

Covert hepatic encephalopathy (CHE), which consists of minimal hepatic encephalopathy (MHE) and grade 1 West Haven Criteria hepatic encephalopathy (HE), is characterized by the presence of mild cognitive impairments particularly in attention, visuospatial coordination, executive functions, and psychomotor speed. It is estimated that the prevalence of CHE in patients with cirrhosis is 30%–85% (Ampuero et al., 2018; Ortiz et al., 2005; Vilstrup et al., 2014). In addition, CHE has significant impact on cirrhotic patients, including declined the work performance and/or increased the traffic violations and accidents (Labenz et al., 2018; Ortiz et al., 2005). Furthermore, CHE is associated with increased progression to overt HE (OHE), which have a negative impact on patient's quality of life (Ampuero et al., 2018; Labenz et al., 2018; Ortiz et al., 2005) and cause a high mortality (Cui et al., 2018). Therefore, testing for CHE in patients with HBV‐related cirrhosis is beneficial to the patients and it is recommended (Vilstrup et al., 2014). Due to its subtle clinical symptoms, diagnosis of CHE mainly depends on specialized neurophysiologic, computerized and paper–pencil tests such as psychometric hepatic encephalopathy score (PHES). These tests seem simple, but they all should be performed by experienced examiners, and the test results are usually affected by the patient's age and literacy (Ortiz et al., 2005; Vilstrup et al., 2014). Owing to the complexity of diagnostic strategies, the subjectivity of evaluations and the lack of sufficient attentions (Patidar & Bajaj, 2015), most CHE patients do not receive timely diagnosis and appropriate treatment and are faced with risk of accidents. Thus, validating a noninvasive and objective method of diagnosis would be beneficial for both selecting therapeutic strategies and prognosis in clinical practice.

Now, a variety of magnetic resonance imaging (MRI) technology has been widely used in HE researches. MRI can identify abnormalities in brain structures and functions in patients with HE. Many researches showed that patients with CHE or OHE had significant alterations in PC, including gray and white matter volume (Chen et al., 2012; Iwasa et al., 2012; Montoliu et al., 2012; Wu et al., 2015), functional connectivity (Chen et al., 2013, 2017; Yang et al., 2018), and diffusion properties (Chen et al., 2017; Lin et al., 2012; Qi et al., 2012). In addition, some researchers found that the alterations of the volume of PC were correlated with the ammonia levels and the extent of cognitive impairment (Chen, Liu, et al., 2017; Montoliu et al., 2012; Wu et al., 2015). Therefore, the alterations of PC might be one of important neuropathological mechanisms of cognitive dysfunction and be relevant to the early diagnosis of CHE.

Radiomics is an auxiliary detection and diagnostic technique that converts medical images into high‐dimensional mineable data. It is intended to develop decision support tools. Radiomic data and available clinical factors can increase the power of the decision support models, which may potentially improve diagnostic and predictive accuracy and evaluation of prognosis for disorders (Gillies et al., 2016). Nowadays, radiomics analysis was not only applied in the tumor field such as classifying tumors (Aerts et al., 2014) and predicting their outcomes (Huynh et al., 2016), but also was used in the nontumor field of Alzheimer's disease (AD) (Feng, Wang, et al., 2018; Li et al., 2019), Parkinson's disease (Wu et al., 2019), attention‐deficit hyperactivity disorder (Sun et al., 2018), and Autism Spectrum Disorder (Chaddad et al., 2017; Heinsfeld et al., 2018). Those studies had shown that objective and quantitative features could potentially provide a new approach to develop classifiers, which may facilitate the individualized diagnostic biomarkers. But currently, CHE has no research use radiomics analysis.

**Figure 1:**
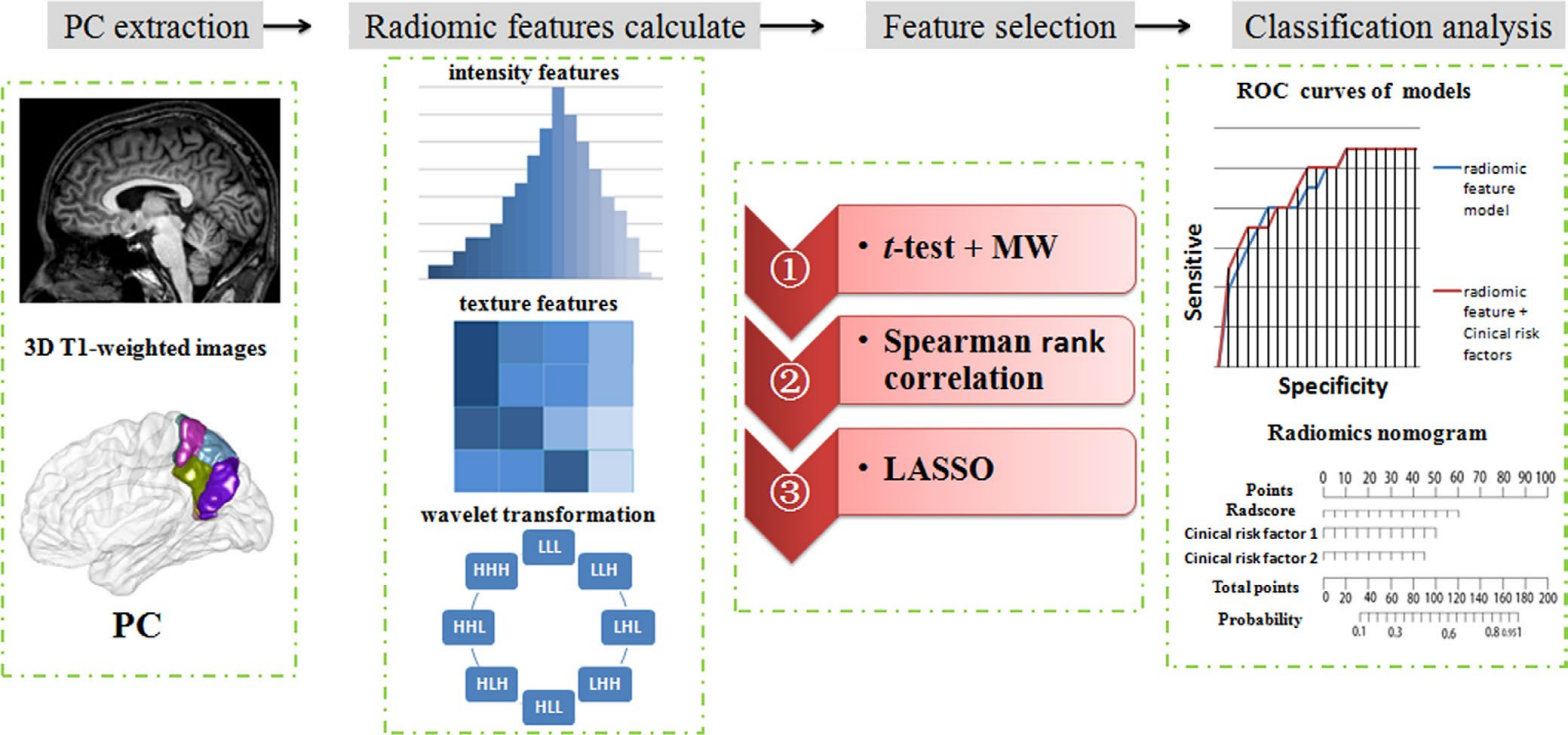
The workflow of data processing

The aim of this study is to extract quantitative features from PC and their subregions and combine with clinical risk factors to develop and evaluate classification models for HBV‐related cirrhotic CHE in a framework of radiomics analysis (Figure 1).

## 2 METHOD

### 2.1 Participants

A total of 106 patients with HBV‐related cirrhosis diagnosed by liver biopsy or clinical criteria were consecutively recruited from February 2018 to June 2019 and written informed consents were obtained from all participants, and this study was approved by the local ethics committee (ethics reference number: 2018–043). Exclusion criteria was followings: (a) current overt HE or other neuropsychiatric disorders; (b) liver malignancy; (c) history of brain surgery; (d) alcohol abuse within 6 months prior to the study; (e) active infection; (f) recent (<4 weeks) gastrointestinal bleeding; (g) metabolic diseases or endocrine diseases (e.g. diabetes mellitus or and thyroid dysfunction); (h) history of taking psychotropic medications; (i) age ≤ 18 years or ≥75; and (j) MRI contraindications.

### 2.2 Paper–pencil testing and diagnosis of CHE

The diagnosis of CHE was made according to the practice guideline of the 14th International Society for Hepatic Encephalopathy (Vilstrup et al., 2014). Patient who showed abnormal scores in the number connection test A (NCT‐A) and digit symbol test (DST) was defined as CHE. The abnormal scores were defined as exceeding the reference value by two standard deviations, referred to the normal value of a domestic expert consensus of China (Xing, 2009). The tests were performed in 1 hr before MRI scan. As a result, participants were divided into CHE group (54 patients) and non‐CHE group (nCHE) group (52 patients).

### 2.3 Clinical staging and laboratory examinations

Data including the Child‐Pugh stage (based on albumin, total serum bilirubin, prothrombin time, and ascites) and venous blood ammonia were obtained within one week prior to MRI scan to assess the severity of liver disease for each subject.

### 2.4 MR imaging

MRI examinations were performed using a 3.0 T MR scanner (Achieva 3.0 T TX; Philips Healthcare) with an eight‐channel head coil for all participants. The three‐dimensional (3D) T1‐weighted images of the brain were acquired for each subject using a rapid gradient echo sequence (TR/TE: 7.8/3.8 ms, TI: 920.8 ms, FOV: 240 mm × 240 mm, acquisition matrix: 240 × 240, flip angle: 8°, slice thickness: 1 mm, 150 sagittal slices and resolution of 1 mm × 1 mm × 1 mm).

### 2.5 Segmentation of PC and their subregions

10 regions of interest (ROI) segmentation of the PC was carried out using the statistical parametric mapping (SPM8, www.fil.ion.ucl.ac.uk/spm/) and the Brainnetome Toolkit (version 3.35; http://atlas.brainnetome.org/) (Feng, Wang, et al., 2018). According to the Human Brainnetome Atlas (based on anatomical and functional connections), each side of PC has 4 subregions including medial area 7 (PEp), medial area 5 (PEm), dorsomedial parieto‐occipital sulcus (PEr), and area 31 (Lc1) (Fan et al., 2016). 10 ROIs (right PC,right PEp, right PEm, right PEr, right Lc1, left PC, left PEp, left PEm, left PEr, and left Lc1) were obtained as follows: First, the 3D T1‐weighted images of each subject were converted into the format that can be used for postprocessing; second, the images were performed skull stripping and normalized to the Montreal Neurological Institute (MNI) space, and the Brainnetome Atlas was also resliced to the standard MNI space; third, bilateral PC and their subregions’ 3D masks were automatically obtained from the resliced brain atlas; finally, point multiplication of the 3D masks and the normalized T1 images were used to get the 10 ROIs.

### 2.6 Quantitative radiomic features calculate

The calculation of radiomic features were carried out by using in‐house MATLAB scripts (http://atlas.brainnetome.org/) (Feng, Wang, et al., 2018). 423 radiomic features were calculated from each ROI. There are 10 ROIs, a total of 4,230 (423 × 10) radiomic features for the further analysis. The features included follows (1) 14 intensity features, which calculated from the histogram of voxel intensity, (2) 22 texture features from the Gray‐Level Co‐occurrence Matrix (GLCM) and 11 texture features from the Gray‐Level Run‐Length Matrix (GLRLM), and (3) 376 post‐Wavelet transformation features we applied wavelet transformation in LLL, LLH, LHL, LHH, HLL, HLH, HHL, and HHH directions.

### 2.7 Statistical analysis

#### 2.7.1 Demographic variables

All demographic and clinical details analyses were performed with R software (version 3.6.0; http://www.Rproject.org), and the threshold of significance was set at a level of *p* < .05. The chi‐square test used to examine differences in gender between the CHE and nCHE, and the two‐tailed independent samples *t* test was hired to assess the other demographic variables.

#### 2.7.2 Feature selection

First, 106 patients with HBV‐related cirrhosis were randomly divided into training set and testing set, with a proportional of 7:3. Thus, 73 patients (37 CHE and 36 nCHE) were randomly selected as the training set for feature selection and classification models construction. The other 33 patients (17 CHE and 16 nCHE) were included in the testing set and only used to test the models. The feature selection steps are described below. First step: the Shapiro–Wilk test was used to check normality, the normally distributed data were analyzed by *t* test, and non‐normally distributed data were analyzed by Mann–Whitney *U* test. A significance threshold of *p* < .05 was applied to each test. Second step: Spearman rank correlation was employed to eliminate high‐dimensional feature redundancy. If the two features were highly correlated (correlation coefficient > 0.9), then excluded one of them. Third step: the using of the least absolute shrinkage selection operator (LASSO). The LASSO is a shrinkage and selection method for linear regression. It minimizes the usual sum of squared errors, with a bound on the sum of the absolute values of the coefficients (Meng et al., 2020). The optimization objective for LASSO is:

$$\min_{\beta }\left(\frac{1}{n}\sum_{i=1}^{n}{\left(y_{i}‐x_{i}\beta^{T}\right)}^{2}+\lambda {\left\|\beta \right\|}_{1}\right)$$

where *x_i_* is the *i*‐th patient's feature vector, $y_{i}$ is the classification variable, β is the weight vector of the linear model, and λ>0 is a penalty term, which controls the value of shrinkage.

The selection step was embedded in a 10‐fold cross‐validation framework to obtain unbiased estimates of classification error and then chose the λ to get the minimum criteria (Varma & Simon, 2006).

#### 2.7.3 Classification models construction

Two classification models were developed to diagnose CHE. First, the logistic regression analysis was employed to construct the radiomics signature model (R model), which only use the radiomic features of the radiomics signature. Then, multivariable logistic regression analysis was used to develop another model (R‐C model), which incorporated the radiomics score (Radscore) and clinical risk factors. A linear combination was applied on radiomics signature to get the Radscore of each subject. The formula is as follows:

$$\mathrm{Radscore}=\beta_{0}+\beta_{1}x_{1}+\beta_{2}x_{2}+\beta_{3}x_{3}+\beta_{4}x_{4}$$

$\beta_{0}$ represents a constant, and there $\beta_{0}=‐0.553$, $\beta_{i}$ is the coefficient of radiomic feature, $x_{i}$ is the value of the feature.

The diagnostic performance of the two models were assessed in the training set by using the area under the curve (AUC) of the receiver operating characteristic curve (ROC), and then, they were evaluated in the testing set. At the same time, Delong test was used to observe whether the models are under‐fitting or over‐fitting.

#### 2.7.4 Development of radiomics nomogram

To provide the clinician with a quantitative tool to predict individual probability of CHE, the radiomics nomogram was built on the basis of the model with the highest predictive efficiency. And the calibration curves of testing set and training set were plotted to assess the calibration of the radiomics nomogram. And the Hosmer–Lemeshow test was employed to assess the goodness of fit of calibration curve for radiomics nomogram (Huang et al., 2016).

#### 2.7.5 Correlation analysis

The correlations among cognitive test scores (NCT‐A and DST), venous blood ammonia, the Child‐Pugh stage, demographic characteristics (age, gender, and education level), and radiomic features were studied via Spearman correlation analysis.

## 3 RESULTS

### 3.1 Demographic characteristics and paper–pencil testing

The demographics, neuropsychological tests, and biochemical parameters of training set and testing set are summarized in Table 1. Compared with the nCHE, CHE spent more time to complete the NCT‐A and had less correct number of DST (*p* < .001). And the venous blood ammonia of CHE was significantly higher than that of nCHE (*p* < .001). More CHE patients had high‐level Child‐Pugh stage both in training and testing set (*p* < .001). There were no significant differences in age, gender and education level between the CHE and nCHE (*p* > .05) in the training set and testing set.

**Table 1:** Demographic and clinical characteristics of all subjects

Protocols	Training set (*n* = 73)			Testing set (*n* = 33)			*p*
	CHE (*n* = 37)	nCHE (*n* = 36)	*p*	CHE (*n* = 17)	nCHE (*n* = 16)	*p*	
Demographics							
Age (year)	57 ± 9.98	53.22 ± 8.99	0.07	57.94 ± 11.98	56.63 ± 11.89	0.885	0.32
Gender (M/F)			0.747			0.304	0.481
Male	25 (34.3%)	23 (31.5%)		11 (33.3%)	13 (39.4%)		
Female	12 (16.4%)	13 (17.8%)		6 (18.2%)	3 (9.1%)		
Education level	7.95 ± 4.26	9.89 ± 3.03	0.051	8.65 ± 2.98	9.43 ± 3.56	0.939	0.869
Laboratory examinations							
NH_3_ (μmol/L)	57.43 ± 44.45	21.36 ± 12.13	<0.001*	63.63 ± 33.81	21.94 ± 12.37	<0.001*	0.716
Child–Pugh stage			<0.001*			0.002*	0.672
A	8 (11.0%)	21 (28.8%)		3 (9.1%)	10 (30.2%)		
B	13 (17.8%)	12 (16.4%)		5 (15.2%)	5 (15.2%)		
C	16 (21.9%)	3 (4.1%)		9 (27.3%)	1 (3.0%)		
Neuropsychological tests							
NCT‐A (s)	63.65 ± 32.00	31.37 ± 7.26	<0.001*	67.91 ± 27.33	36.45 ± 13.77	<0.001*	0.400
DST	24.51 ± 11.59	38.53 ± 9.18	<0.001*	21.94 ± 8.41	35.94 ± 10.42	<0.001*	0.298

Note:

Values are expressed as mean ± *SE*.

Abbreviations: CHE, cirrhotic patients with covert hepatic encephalopathy; DST, Digit Symbol Test; nCHE, cirrhotic patients without hepatic encephalopathy; NCT‐A, Number Connection Test A; NH_3_, the venous blood ammonia.

* Significant differences between groups.

### 3.2 Feature selection results

**Figure 2:**
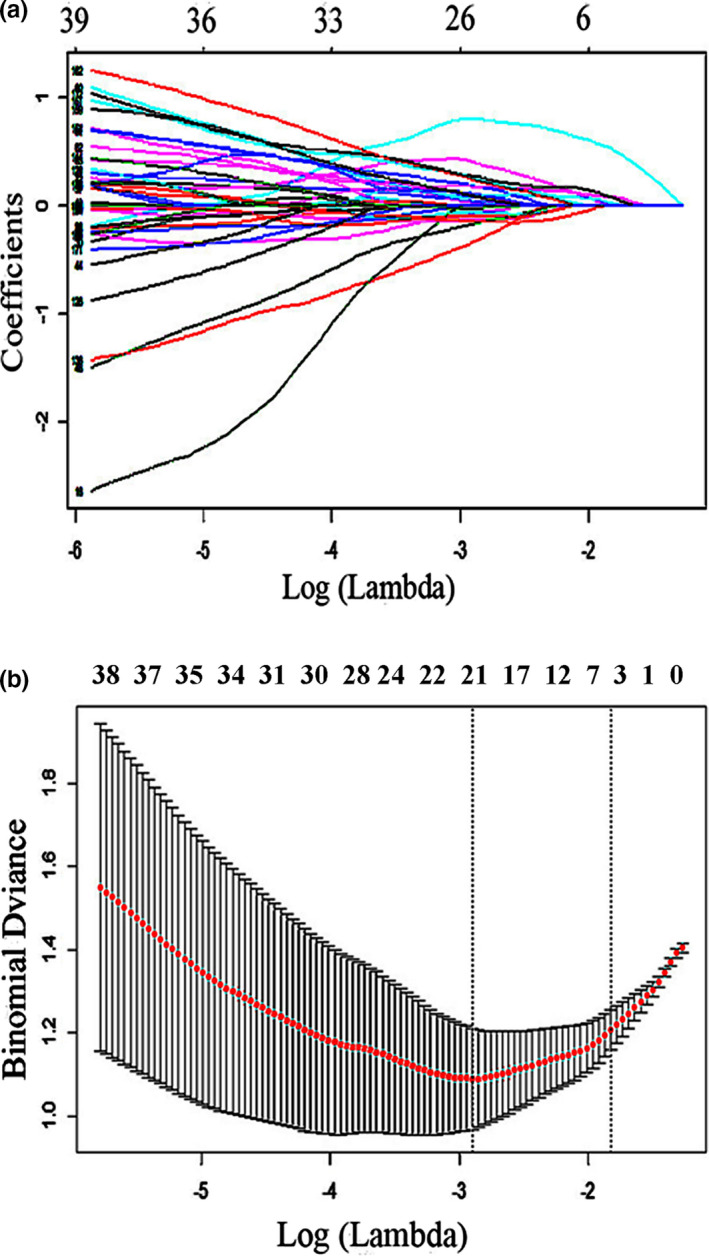
Plot of: (a), coefficients‐lambda; (b), error‐lambda. A 10‐fold cross‐validation was applied with the regularization parameter (λ) of the LASSO regression model and was selected when the deviance was minimal (a). Coefficients are plotted against the log (λ) sequence. Ultimately, four nonzero coefficients were selected (b)

346 features remained by using *t* test and Mann–Whitney *U* test, including 247 features (71.39%) of the right ROIs and 99 features (28.61%) of the left ROIs; 178 features were remained by using Spearman rank correlation method; 4 features were remained as the radiomics signature by using LASSO, the features as follows: (a) right PEp_Variance HLH, (b) right Lc1_Median HLL, (c) right Lc1_GrayLevelNonuniformity (GLN) LLL, and (d) right Lc1_Informational Measure of Correlation 1 (IMC1) LLL. The coefficients‐lambda graph and error‐lambda graph are shown in Figure 2a,b.

### 3.3 Classification models

**Figure 3:**
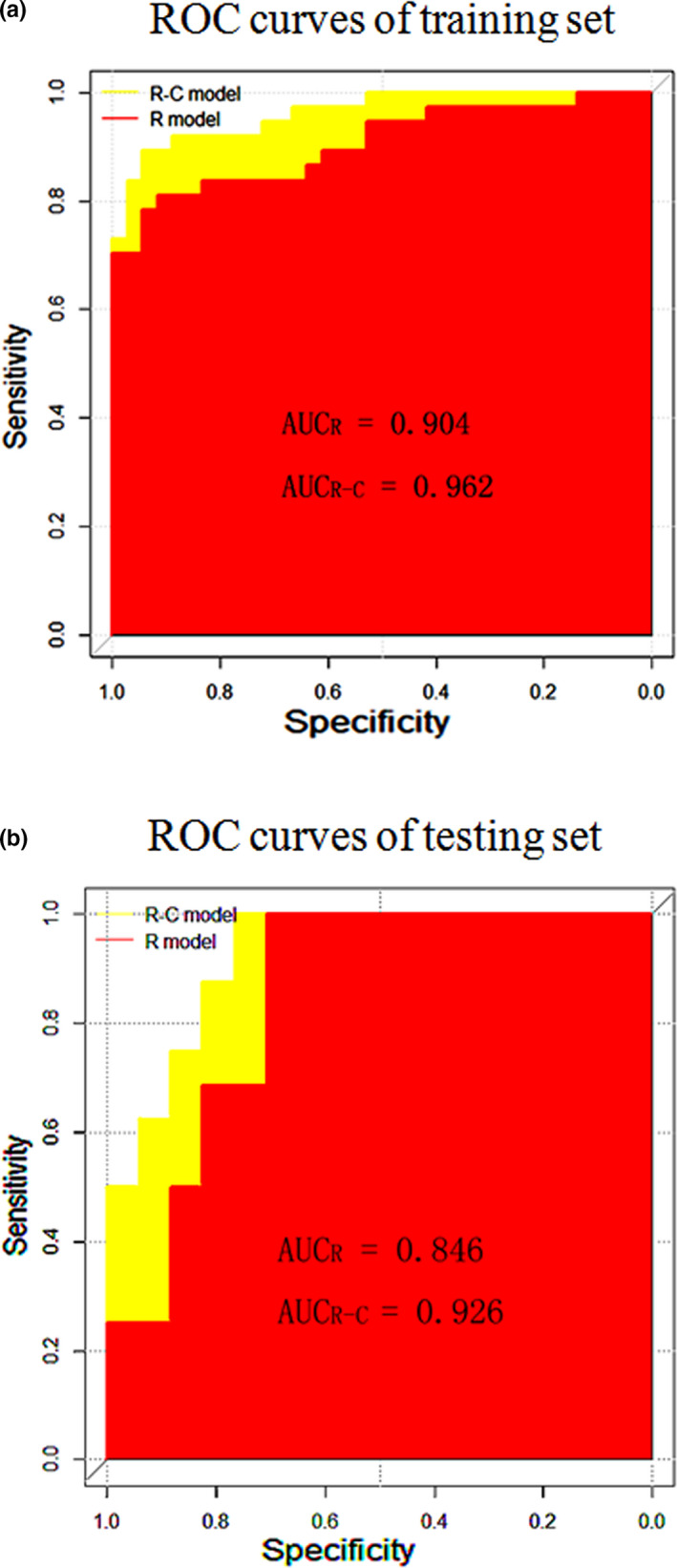
Graph shows the ROC curves of: (a), the models of training set; (b), the models of testing set. R model: the model based on radiomics signature. R‐C model: the model based on radiomics signature and clinical risk factors

**Figure 4:**
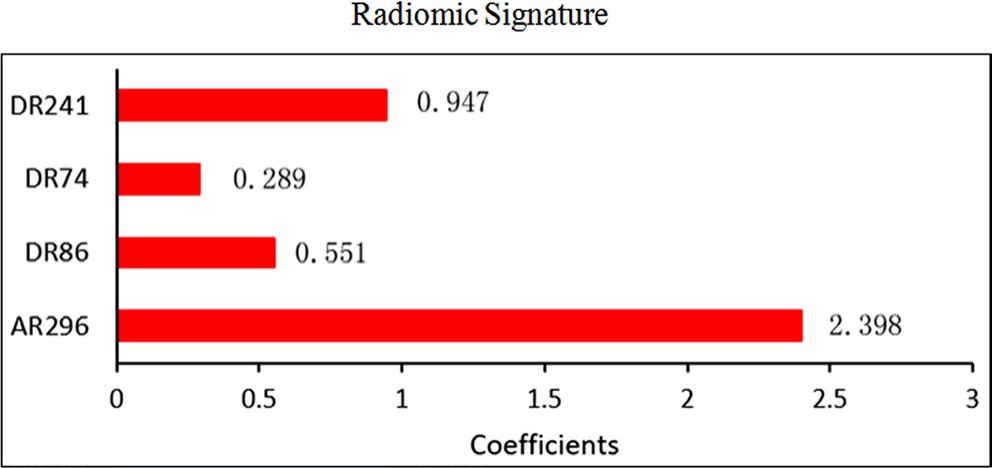
Graph shows radiomics signature. There are 4 features and their coefficients value (Radscore coefficient). AR296, right PEp_Variance HLH. DR241, right Lc1_Median HLL. DR86, right Lc1_GrayLevelNonuniformity (GLN) LLL. DR74, right Lc1_Informational Measure of Correlation 1 (IMC1) LLL

The results showed that the diagnostic performance of R‐C model was superior than the R model, as shown in Table 2. The R‐C model had higher AUC (0.926 95% CI, 0.780–0.988) and specificity (0.765) and with the same accuracy (0.848) and sensitivity (1.000) than the R model, in the testing set. The AUC, accuracy, sensitivity, and specificity in testing set of the R model were 0.846 (95% CI, 0.678–s0.947), 0.848, 1.000, and 0.706, respectively. The ROC curve of models is shown in Figure 3a,b, and the coefficients value of Radscore coefficient for each radiomic feature is shown in Figure 4.

**Table 2:** Diagnostic performance of the different models for predicting CHE

Methods	Training set (*n* = 73)				Testing set (*n* = 33)				*p*
	AUC (95% CI)	ACC	SEN	SPE	AUC (95% CI)	ACC	SEN	SPE	
R model	0.904 (0.812–0.960)	0.836	0.784	0.944	0.846 (0.678–0.947)	0.848	1.000	0.706	0.455
R‐C model	0.962 (0.889–0.993)	0.917	0.892	0.944	0.926 (0.780–0.988)	0.848	1.000	0.765	0.463

Abbreviations: ACC, accuracy; AUC, area under the curve; CI, confidence interval; R model, the model based on radiomics signature; R‐C model, the model based on radiomics signature and clinical risk factors; SEN, sensitivity; SPE, specificity.

### 3.4 The radiomics nomogram and the calibration curve

**Figure 5:**
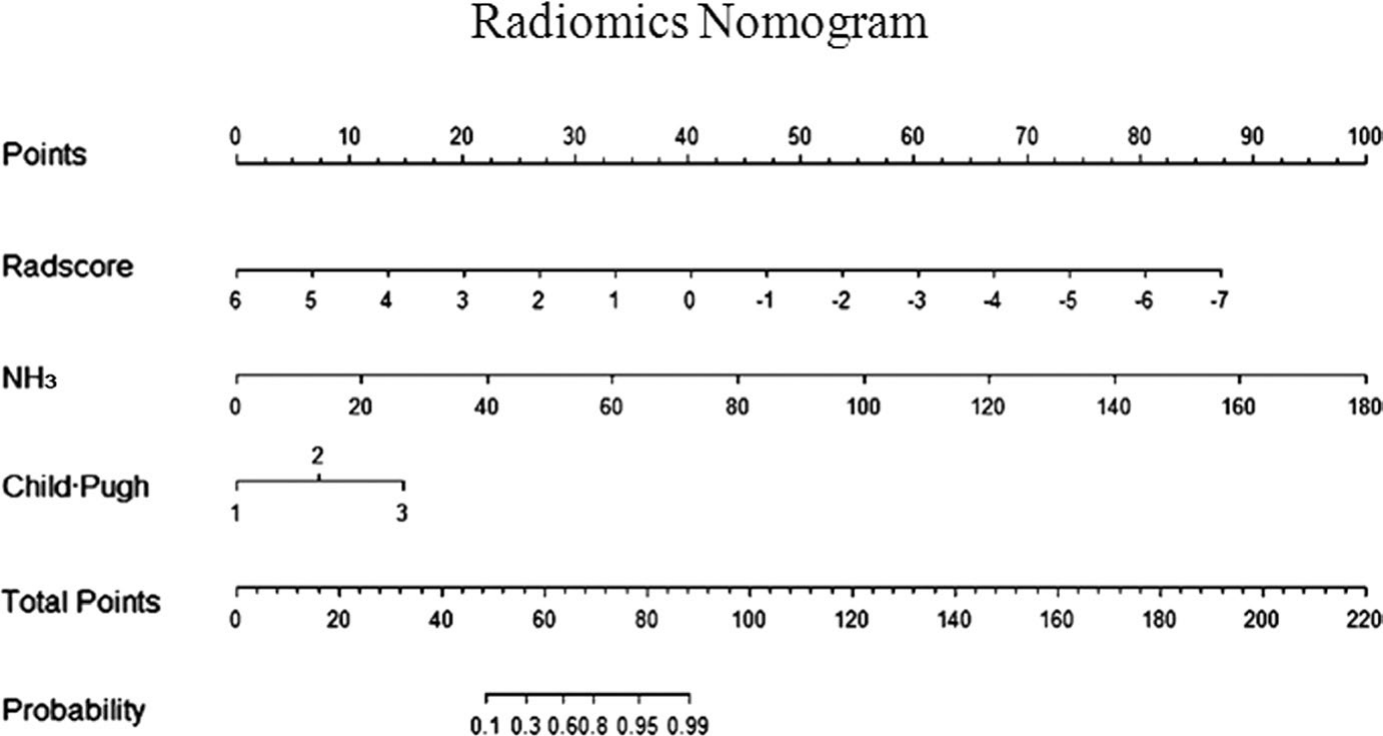
Graph shows the radiomics nomogram. The radiomics nomogram was developed in the multivariable logistic regression classifier of training set data, which combines three items: Radscore, NH_3_, and Child–Pugh stage. Radscore, radiomics score; NH_3_: venous blood ammonia

**Figure 6:**
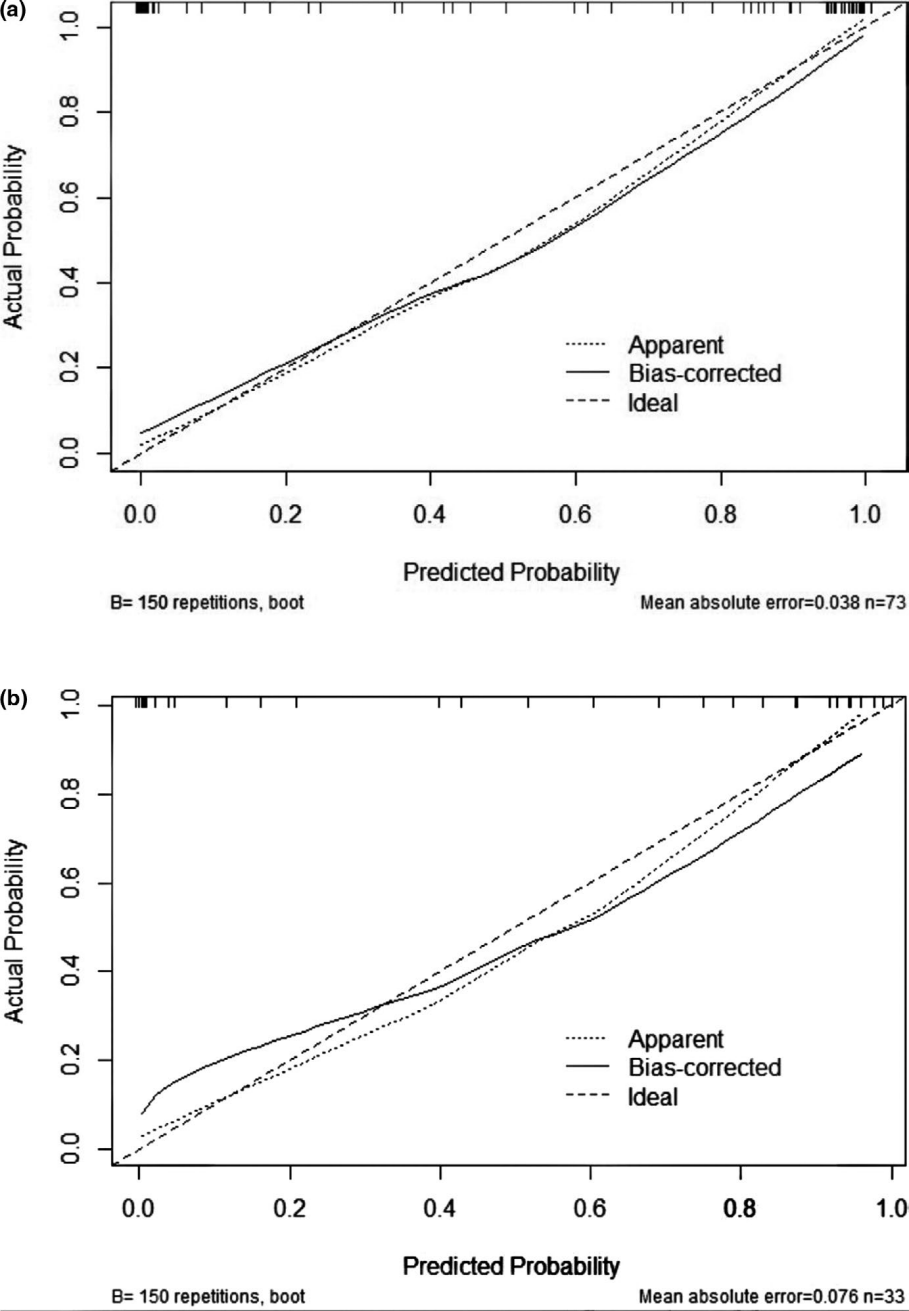
Calibration curves of radiomics nomogram‐based prediction in the training set (a) and testing set (b). The calibration curves represent the calibration of the nomogram based on agreement between the predicted risk of CHE and actual CHE. A close fit between the dotted and solid lines indicates good predictive accuracy of the nomogram

The R‐C model with the highest predictive efficiency was developed and presented as the radiomics nomogram (Figure 5). The calibration curve for the radiomics nomogram was tested by the Hosmer–Lemeshow test, and the results showed no significant difference between the calibration curves and a perfect fit for predicting CHE, whether in the training set (*p* = .850) or the testing set (*p* = .475) (Figure 6).

### 3.5 Correlations analysis

**Figure 7:**
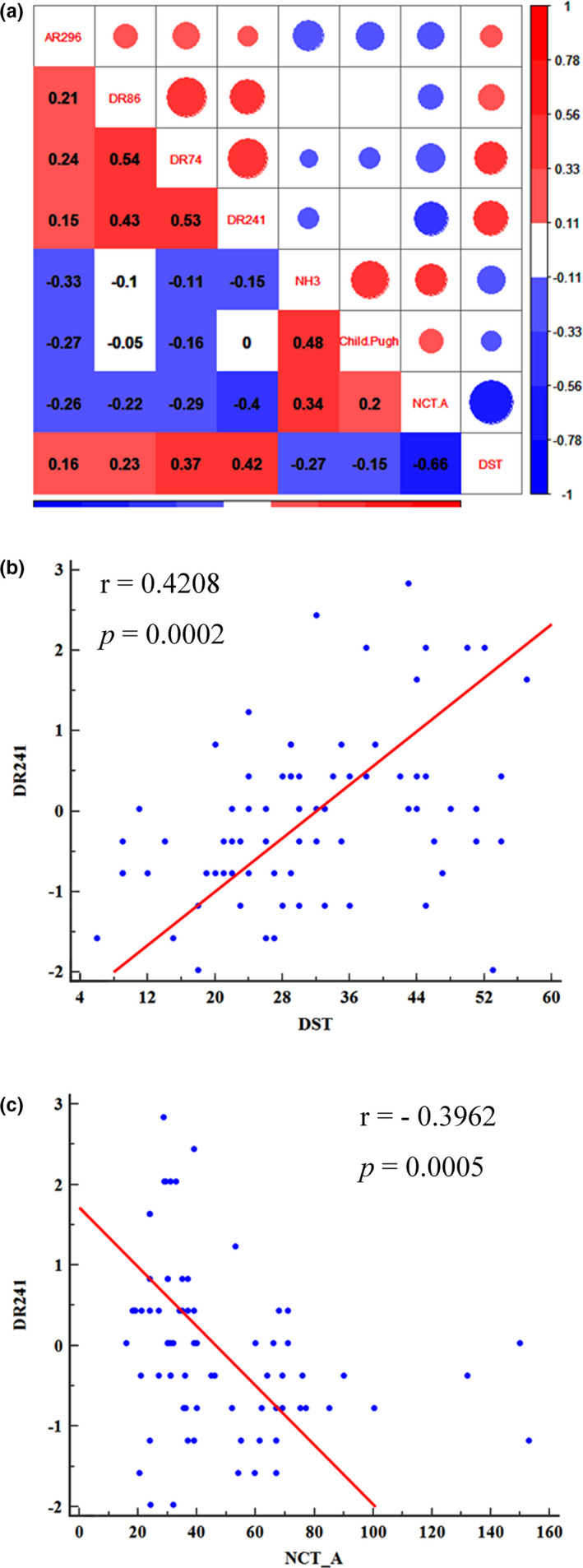
Graph shows the correlations analysis. (a), the correlations analysis of the radiomic features, venous blood ammonia, Child–Pugh stage, and paper–pencil testing (NCT‐A and DST) of all the cirrhotic patients; (b), positive correlations between the typical feature (DR241, right Lc1_Median HLL) and DST score; (c), negative correlations between the typical feature (DR241, right Lc1_Median HLL) and NCT‐A score

Spearman correlation analysis suggested that all the 4 radiomic features (right PEp_Variance HLH, right Lc1_Median HLL, right Lc1_GLN LLL, right Lc1_IMC1 LLL) are positively correlated with DST scores and negatively correlated with NCT‐A scores (Figure 7a). The correlation coefficients of the typical feature (DR241, right Lc1_ Median HLL) with DST score and NCT‐A score are 0.42 and −0.40, respectively (Figure 7b,c).

## 4 DISCUSSION

In our study, we identified significantly different radiomic features in PC between CHE and nCHE. After LASSO, we finally found 4 radiomic features including Variance, Median, GLN, and IMC1 which showed significant differences in PC of CHE when contrasted to nCHE. PC must have great changes in CHE. As a result of liver dysfunction, subsequently concentrations of the ammonia, reactive oxygen, and nitrogen, etc. rise in the blood. Those chemicals cross the blood–brain barrier, then effect on many signal transduction pathways (Wang et al., 2015) and trigger astrocyte swelling (Mínguez et al., 2006) and even cellular senescence (Görg et al., 2014). As we know, Median and Variance are measures of voxel intensity values in brain images and GLN 1 and IMC1 represent the distributions of voxel values. The abnormalities of cerebral cells such as astrocyte swelling even cellular senescence lead to changes of the voxel intensity and its special distribution feature and pattern in PC, and cause the significantly changed radiomic features in CHE.

At the same time, our study showed radiomic features (Median, Variance, GLN 1, and IMC1) in CHE correlated with NCT‐A, DST. This was coincident with previous studies, they found the functional connectivity strength, and diffusion kurtosis imaging metrics and cortical thickness of PC were correlated with PHES in cirrhotic patients without OHE (Chen, Lin, et al., 2017; Chen, Liu, et al., 2017; Wu et al., 2015) as well. PC is a major association area, which has wide‐spread connectivity with both cortical and subcortical structures. It was proven to be a critical area with multimodal and integrative functions including consciousness, visuospatial imagery, episodic memory retrieval, and self‐processing operations (Cavanna & Trimble, 2006; Margulies et al., 2009). The underlying pathological changes of PC may cause impaired psychomotor speed, visual scanning efficiency, attention, and other functions, that is why patients with CHE spend more time to complete NCT‐A. And the dysfunction of cognitive processing speed, visual perception, and working memory (Weissenborn, 2008) lead to the lower DST in CHE.

More interesting, most of our significant radiomic features of CHE were from the right PC, and this kept in line with previous studies revealing right PC was seem to have more obvious changes than the left side (Montoliu et al., 2012; Qi et al., 2013; Wu et al., 2015). Right PC recall memories more (Freton et al., 2013) and have more prominent characteristics about people's social interactions (Petrini et al., 2014). This can explain CHE patients have declined work performance (Labenz et al., 2018; Ortiz et al., 2005) and life quality (Ampuero et al., 2018; Labenz et al., 2018; Ortiz et al., 2005). We strongly believe abnormalities of PC especially the right one can provide a new potential image marker for CHE.

For the radiomics model, previous CHE studies (Chen, Liu, et al., 2017; Wu et al., 2015) only revealed the abnormalities of PC but they did not construct a classifier to prove its diagnostic power. In our research, firstly we constructed the R model, it had a good diagnostic performance (AUC 0.846) to differentiate the CHE from nCHE. To our understanding, no clinical risk factors were applied to form a classifier for CHE. Further, associated the clinical risk factors (venous blood ammonia and Child‐Pugh stage), we made the R‐C model. This improved the diagnostic performance (AUC 0.926). Radiomics classifier was proven to be a powerful diagnostic tool; it successfully verified the AD by radiomic features of hippocampus (Feng, Wang, et al., 2018) and corpus callosum (Feng, Chen, et al., 2018). One recent radiomics study used hippocampus to recognize autism spectrum disorder (Chaddad et al., 2017) with high diagnostic performance (AUC 76.80%). Compared with other medical image‐based researches, radiomics extracts high‐dimensional features. Those data are quantitative and objective. It can improve predictive accuracy compared with traditional ways (Gillies et al., 2016). Further, using the R‐C model we developed the radiomics nomogram it reached a satisfactory result when applied to the testing set. This nomogram could be conveniently used to the individualized prediction of CHE in patients with cirrhosis.

Nevertheless, there are several limitations in this study. First, due to the small sample size, the classification performance may show high sensitivity, using multicenter data sets is a solution for challenges of lager sample size in the future. Second, PC may also have alterations in function and structure, such as diffusion properties and functional connectivity. Combination of other MRI‐based markers, radiomic features, and other clinical factors is needed for future researches. Third, in our study, the CHE patients were diagnosed according to the expert consensus on diagnosis and treatment of hepatic encephalopathy in China (Xing, 2009). The cirrhotic group without CHE might be underestimated. Finally, healthy group was not included in this study due to the aim of our study. Which was to develop radiomics‐based classification models for differentiate CHE from cirrhotic patients without CHE. It is an interesting attempt to explore whether the radiomic features of PC show similar or different changes between cirrhotic patients without CHE and healthy group. This requires further research in the future.

## 5 CONCLUSION

In conclusion, our results highlight the importance of radiomic features of PC subregions, especial the right PC; this can be regarded as a potential image marker of CHE. The radiomics nomogram that incorporates the radiomics signature and clinical risk factors may facilitate the individualized diagnosis, which can be conveniently used to identify the cirrhotic patients with CHE.

## CONFLICT OF INTEREST

The authors declare that they have no conflict of interest.

## AUTHOR CONTRIBUTIONS

Wei Zhang and Wei‐jia Zhong contributed equally to study concept and design, revising manuscript, and study supervision. Sha Luo and Zhi‐Ming Zhou were equally responsible for data collection, data analysis, drafting the manuscript, and study design. Da‐Jing Guo, Chuan‐Ming Li, and Huan Liu contributed to the statistical analysis and revising manuscript. Wei Zhang obtained the funding to support this work. Xiao‐Jia Wu, Shuang Liang, Xiao‐yan Zhao, Ting Chen, Dong Sun, and Xin‐Lin Shi involved in MRI and clinical data acquisition. All authors approved the final version of the manuscript.

### Peer Review

The peer review history for this article is available at https://publons.com/publon/10.1002/brb3.1970.

## Data Availability

The data that support the findings of this study are available from the corresponding author upon reasonable request.
